# Prevalence and factors associated with probable anxiety disorders among elderly persons living with HIV at Mulago ISS clinic: A cross-sectional study

**DOI:** 10.1371/journal.pone.0329111

**Published:** 2025-08-01

**Authors:** Anthony Muyunga, Kevin Ouma Ojiambo, Janet Nakigudde, Jovan Mugerwa, Benard Owori, Kevin Naturinda, Brian Mikka, Janet Peace Babirye, Namutale R. Nalule, Isaac Samuel Kintu, Enos Kigozi, Caroline Birungi

**Affiliations:** 1 Clinical Epidemiology Unit, Department of Internal Medicine, School of Medicine College of Health Sciences, Makerere University, Kampala, Uganda; 2 Reach Out Mbuya Community Health Initiative, Kampala, Uganda; 3 Uganda National Institute of Public Health (UNIPH), Ministry of Health, Uganda; 4 Department of Psychiatry, School of Medicine, Makerere University College of Health Sciences, Kampala, Uganda; 5 Infectious Diseases Institute, College of Health Sciences, Makerere University, Kampala, Uganda; 6 Mulago Immune Suppression Syndrome Clinic, Mulago National Referral Hospital, Kampala, Uganda; Johns Hopkins University School of Medicine, UNITED STATES OF AMERICA

## Abstract

**Introduction:**

Human Immunodeficiency Virus/Acquired Immunodeficiency Syndrome (HIV/AIDS) is a major public health concern globally. Due to advancements in Anti-Retroviral Treatment (ART) therapy, more people with HIV are living longer with about 1.4 million infected people in Uganda. Anxiety disorders are often unrecognized and undetected in older persons living with HIV (PLWH) yet they impair an elderly person’s physical health and decrease the ability to perform daily activities.

**Objective:**

To determine the prevalence and factors associated with probable anxiety disorders among elderly PLWH at Mulago Immune Suppression Syndrome (ISS) clinic.

**Methods:**

A cross-sectional study was conducted at Mulago ISS clinic among 273 systematically selected participants living with HIV/AIDS on antiretroviral therapy for at least 6 months between April and May 2024. Interviews were conducted using the Generalized Anxiety Disorder 7-item (GAD-7) screening tool to help identify individuals who may be at risk for anxiety disorders and structured questionnaires for socio-demographics, and psychological factors. Drug and clinical factors data were extracted from records, entered into Epidata, and later to STATA version 17 for analysis. Prevalence was reported as a percentage and modified Poisson regression analysis was used to determine the factors associated with anxiety disorders.

**Results:**

We enrolled 273 participants with a median age (Interquartile range) was 56 (52, 61.5) years. 54.9% were females, 56.8% didn’t have a partner and 53.8% were employed. The prevalence of probable anxiety disorders was 16.8% (95% CI 12.5–21.6). Employment status (aPR- 2.113, 95% CI 1.252–3.567), family history of mental health disorder (aPR-2.041, 95% CI 1.228–3.394), stigma (aPR-2.564, 95% CI 1.544–4.257) and family support (aPR-2.169, 95% CI 1.272–3.699) were significantly associated with having probable anxiety disorders.

**Conclusion:**

One in every six elderly persons living with HIV may have a probable anxiety disorder. Being unemployed, having a family history of mental health disorders, having stigma and having inadequate family support were significantly associated with having a probable anxiety disorder. Healthcare workers should provide comprehensive anxiety screening and patient-centered care for elderly persons with HIV. At the same time, the government develops financial empowerment strategies and supports mental health through family groups, and public campaigns to reduce HIV stigma and educate families on effective support.

## Introduction

HIV/AIDS is an infectious disease caused by the human immunodeficiency virus and is a major public health issue globally. In 2021, about 38.4 million people were estimated to be living with HIV worldwide, of which 680,000 died from AIDS-related illnesses [1]. Sub-Saharan Africa has more than two-thirds of all people living with HIV (PLWH) globally making it the hardest-hit region in the world [2]. The number of PLWH 50 years and above increased substantially from 8% in 2000 to 16% in 2016 and is expected to increase [3]. In 2016, 80% of PLWH 50 years and above lived in low and middle-income countries (LMICs), with Eastern and Southern Africa containing the largest number [3]. The burden of HIV in Uganda is still high with about 1.4 million infected people [4]. Among adult PLWH in Uganda, those aged 50 and above now comprise 60% of the population [5]. Living with HIV can be emotionally challenging, with many individuals experiencing anxiety disorders at a higher rate than the general population. HIV-related stigma, which can persist even as individuals age, can contribute to anxiety. Older PLWH may still face discrimination or fear of disclosure of their HIV status, leading to heightened anxiety and social isolation [6]. Ageing can sometimes be associated with social isolation, particularly if friends or partners have passed away or if one’s social network has diminished. Isolation can contribute to feelings of anxiety and depression [7]. Self-neglect resulting from anxiety may further impact their physical and mental health, ultimately reducing their quality of life and overall well-being.

Although literature recognizes anxiety disorders among the elderly population [8], there is limited information on the prevalence and factors associated with anxiety among elderly PLWH in Uganda. Most studies focus on younger groups, neglecting the unique psychological challenges faced by older individuals. The study intended to provide essential insights for healthcare providers, policymakers, and relevant stakeholders and offer a foundation for developing targeted interventions and support systems within Uganda’s broader HIV care framework. As the ageing population of individuals with HIV grows, understanding the factors associated with anxiety disorders among this demographic is vital for providing comprehensive and effective healthcare [9]. This study aimed to understand the prevalence and factors associated with probable anxiety disorders among this demographic.

## Methods

### Study design

A cross-sectional study was conducted with both descriptive and analytical components employing quantitative methods of data collection.

### Study setting

The study was conducted at Makerere University Joint AIDS Program (MJAP) Mulago ISS clinic among people receiving routine ART care services between April and May 2024. Mulago ISS clinic is the largest HIV clinic in Uganda serving about 17000 PLWH with about 4500 persons aged 50 years and above. Mulago ISS Clinic is a private not for profit Non-Government Organization that provides free comprehensive HIV/AIDS services in Mulago Hospital. The clinic is located along Upper Mulago Hill Road within Kawempe South division in Kampala District.

HIV/AIDS clinical services are provided to patients on weekdays except for public holidays. Services offered include HIV testing, counselling, elimination-of-mother-to-child-transmission services, gender-based-violence services, integrated TB/HIV, integrated HIV/hypertension, laboratory testing, ART drug refills, key populations and priority population services, differentiated service delivery models and HPV screening, mental health screening and management among others. The clinic was chosen as it is centrally located and serves persons from different economic and social lifestyles within the hospital and surrounding communities. The facility offers free services hence accessible for all.

### Study population

PLWH aged 50 years and above were considered elderly due to factors such as reduced immune response to ART, increased mortality risk, higher risk of comorbidities, and early onset of geriatric syndromes [10].

All participants aged 50 years and above with HIV/AIDS who were receiving routine care at Mulago ISS clinic between April and May 2024 and fulfilled the eligibility criteria of the study.

### Eligibility criteria

PLWH aged 50 years and above and on ART for 6 months and above, received routine care at the Mulago ISS clinic between April and May 2024 and provided informed consent to participate in the study.

Patients who at the time of conducting the study were unable to withstand the interview process were excluded from the study.

In HIV programs, patients are considered clinically stable after maintaining ART for at least six months. This period allows for the assessment of adherence, viral suppression, and the management of any initial side effects [11].

### Sample size determination and sampling procedure

The sample size was estimated using Kish Leslie’s formula [12,13]. Considering the prevalence value of 20.2% from a study conducted by Opio and colleagues [14]. This resulted in a sample size of 273 considering a 10% non-response rate.

A systematic random sampling technique was used to select 273 study participants. Every third participant was selected from a sampling frame based on patient orders when coming to the clinic for follow-up appointments. The first participant was randomly selected from the first three study participants using simple random sampling by writing numbers 1–3 on pieces of paper and randomly selecting one and then data was collected and continued for every third study participant. In case the third was not eligible, the next participant was considered.

### Study variables

We collected data on socio-demographics including age, sex, marital status, occupation, monthly income, level of education, religion and home ownership, clinical factors comprised of opportunistic infections, World Health Organization (WHO) staging, recent viral load, ART regimen and duration on ART. Psychological factors were familial history of mental health illness, disclosure status, previous history of mental health disorder, stigma, family support and ART adherence. We also gathered information on alcohol and substance use, smoking, hypertension, diabetes, tuberculosis, previous mental health history and mental health treatment.

The outcome variable was probable anxiety disorder defined as the presence of clinically significant anxiety symptoms which was assessed and measured using a GAD-7 tool. Originally designed as a screening tool for generalized anxiety, the GAD-7 also performs well in detecting three other common anxiety disorders: Panic Disorder, Social Anxiety Disorder, and Post-Traumatic Stress Disorder, as well as any other anxiety disorder. When using a GAD-7 score cut-off of ≥10, its performance as a screening tool is as follows:

sensitivity of 89%,74%, 72%, 66% and 68%, specificity of 82%,81%, 80%, 81% and 88% and a positive likelihood ratio of 5.1, 3.9, 3.6 and 5.5 for generalized anxiety disorder, panic disorder, Social anxiety Disorder, Post-Traumatic Stress Disorder and Any anxiety disorder respectively [15,16].

GAD-7 is a Likert scale tool with 7 questions assessing how often a person has been bothered by these problems on the scale in two weeks. Persons who scored 10 and above were considered to have anxiety disorders. The GAD-7 tool has not been used in Uganda in a similar setting for the elderly. A cut-off score of ≥10 yields a sensitivity of 89% and a specificity of 82% for GAD [17]. The reliability coefficient of Cronbach’s α for the GAD-7 scale among older adults living with HIV from the Kenyan coast was 0.74 [18]. However, in this study, the reliability coefficient was 0.92.

### Data collection and management

Participants were given a unique identification number just after consenting to participate in the study before the interview. Data was collected by the principal investigator (PI) and two registered nurses. The questionnaires were pretested on ten eligible participants to assess their feasibility and consistency. The recruitment period started on 30/04/2024 and ended on 17/05/2024.

Stigma was assessed using a 13-HIV stigma scale with a reliability coefficient of 0.83 [19]. In this study, however, it had a Cronbach’s alpha of 0.87. Family support was assessed using the family support scale for elderly people [20] and had a reliability coefficient of 0.96 in the study. The ART regimen and duration of ART regimen data for clinical variables were extracted from patient files. The questionnaire and data tools were in both Luganda (local language) and English languages.

As clients came for their routine visits at the clinic, at the triage desk, they were assessed for eligibility by the research assistant. Participants were explained the study and allowed to make an informed decision and sign a consent form. After signing the consent, the participant was recruited for the research. The PI or research assistant administered the questionnaire either in English or Luganda depending on the participant’s preference which contained socio-demographics, GAD-7, 13-stigma scale, family support scale for the elderly, data abstraction tool for clinical characteristics and psychological characteristics questionnaire.

Data collection tools were checked for completeness by the principal investigator at the dispensing point where participants picked their ART refills. All raw data was stored securely in password-protected laptops, and an external hard drive and would only be accessible to the study team. Double data entry was performed using Epidata version 4.6 to minimize errors. The data was cleaned and then exported to STATA version 14.0 statistical software for analysis.

### Statistical analysis

We summarized the continuous variable (Age) using the median and Interquartile range (IQR) and proportions were used to summarize categorical variables. The prevalence of anxiety disorders among elderly PLWH was reported as a proportion with a 95% confidence interval. Prevalence was also reported for the different sub-populations.

We used the modified Poisson regression model (through Generalized linear models Identity log and family Poisson) with robust standard errors for analysis at both the bivariable and multivariable levels. The modified Poisson technique with robust standard errors was used instead of logistic regression, a more popular technique for a binary outcome because the prevalence of the outcome in this study was high [21]. Where the prevalence of the outcome is not rare, that is, above 10%, the odds ratios produced from a logistic regression model overestimate the effects of the covariates on the outcome [22]. However, the prevalence ratios produced from the modified Poisson regression model are a better alternative for approximating the risk ratio [23].

At the bivariate level, we selected variables associated with a probable anxiety disorder (Outcome variable) with p-values less than 0.2 for inclusion in the multivariable analysis. The assumptions of collinearity and overdispersion were tested and neither was observed. Stepwise elimination was then used to select variables suitable for the final model and only variables with p < 0.o5 were considered. Potential confounding was also assessed using a relative percentage difference of >10% between the adjusted model and the basic model. All tests were two-sided and statistical significance was tested at a 95% level of significance and variables with a p-value less than 0.05 were considered statistically significant.

### Ethical consideration

Ethical clearance was obtained from Makerere University-School of Medicine Research and Ethics Committee (Mak-SOMREC) approval number Mak-SOMREC-2024–878. Informed written consent was also obtained from all study participants. Participants who were found to screen positive for anxiety disorders were referred to clinicians and counsellors for management.

## Results

### Socio-demographic and clinical characteristics of participants

The study participants had a median (IQR) age of 56 (52, 61.5) years. 54.9% (n = 150) were female, regarding marital status 56.8% (n = 155) had no partner. The highest education attained was mostly primary 58.6% (n = 160) and 53.8% (n = 147) were employed, of which 66.0% (n = 97) were self-employed. Monthly income was mostly less than 500,000 Uganda shillings (56.9, n = 82). (Table 1).

**Table 1:** Socio-demographic and clinical characteristics of study participants, N = 273.

Variable	Category	Measure
Age, median (IQR)		56(52,61.5)
Sex, n (%)	Male	123(45.2)
	Female	150(54.9)
Marital Status, n (%)	Has partner	118(43.2)
	No partner	155(56.8)
Education status, n(%)	Primary and below	160(58.6)
	Secondary and above	113(41.4)
Employment status, n (%)	Unemployed	126(46.2)
	Employed	147(53.8)
Source of Income, n (%)	Formal employment	50(34)
	Self-employment	97(66)
Monthly Income, UGX, n (%)	Less than 500,000	82(56.9)
	500,000–1,000,000	52(36.1)
	Above 1,000,000	10(6.9)
Religion, n (%)	Muslim	45(16.5)
	Catholic	88(32.2)
	Anglican	96(35.2)
	Pentecostal	35(12.8)
	Other^*^	9(3.3)
Own a house, n (%)	No	91(33.3)
	Yes	182(66.7)
Opportunistic infections, n (%)	No	252(92.3)
	Yes	21(7.7)
Current Viral load, n (%)	Suppressed	267(97.8)
	Unsuppressed	6(2.2)
Current WHO Stage, n (%)	Stage1	244 (89.4)
	Stage2	20 (7.3)
	Stage3	9 (3.3)
Current ART regimen, n (%)	First line	249 (91.2)
	Second line	22 (8.1)
	Third line	2 (0.7)
Duration of Current regimen, n (%)	less than 3 years	122 (44.7)
	3 years and above	151 (55.3)
Has a comorbidity^¶^, n (%)	No	162 (59.3)
	Yes	111 (40.7)
Alcohol consumption, n (%)	No	216 (79.1)
	Yes	57 (20.9)
Smoking, n (%)	No	261 (95.6)
	Yes	12 (4.4)
HIV disclosure status, n (%)	No	20 (7.3)
	Yes	253 (92.7)
Has a treatment supporter, n (%)	No	116 (42.5)
	Yes	157 (57.5)
Had a previous history of mental health disorder, n (%)	No	253 (92.7)
	Yes	20 (7.3)
Adherence, n (%)	less than 2 doses	235 (86.1)
	2 or more doses	38 (13.9)
Family history of mental health disorder, n (%)	No	218 (79.9)
	Yes	55 (20.1)
Has Stigma, n (%)	No	234 (85.7)
	Yes	39 (14.3)
Family support, n (%)	Inadequate	72 (26.4)
	Adequate	201 (73.6)

(IQR) Interquartile range, (n) sample size, (%) percentage, UGX- Uganda shillings,

* Others (Seventh day Adventists, Traditionalists), WHO- World Health Organization, ART- Anti-Retroviral Treatment,

¶ Comorbidity (HTN-Hypertension, DM-Diabates Mellitus, CVD-Cardio Vascular Disease, TB-Tuberculosis) HIV-Human Immunodeficiency Virus,

92.3% of the study participants (n = 252) had no opportunistic infections, 97.8% (n = 267) had a suppressed viral load, 89.4% (n = 244) were in stage one, 59.3% (n = 162) did not have comorbidity, 79.1% of participants did not consume alcohol (n = 216) and 95.6% (n = 261) were nonsmokers. (Table 1).

92.7% of the study participants had disclosed their HIV status (n = 253), 57.5% (n = 157) had a treatment supporter, 92.7% had no previous history of mental health disorder (n = 253), 86.1% (n = 235) had missed less than 2 doses, 79.9% (n = 218) had a family history of mental health disorders, 85.7% (n = 234) had no stigma, and 73.6% (n = 201) had adequate family support as indicated (Table 1).

### Prevalence of probable anxiety disorders

The overall prevalence of probable anxiety disorders was 16.8% (95% CI 12.5–21.6). The prevalence of probable anxiety disorders was similar among all categories of socio-demographic characteristics. Although prevalence of probable anxiety disorders among Pentecostals 28.6% (95% CI 15.9–45.8) was higher however not significantly different from other religions. (Table 2)

**Table 2:** Prevalence of probable anxiety among elderly PLWH, N = 273.

Variable	Category	N	Anxiety disorders, n (%)	95% CI
Overall prevalence^#^(Probable anxiety disorders)		273	46 (16.8)	12.5 - 21.6
Age Categorized	less than 56	124	21 (16.9)	11.3 - 24.7
	56 & above	149	25 (16.8)	11.6 - 23.7
Sex, n (%)	Male	123	22 (17.9)	12.0 - 25.8
	Female	150	24 (16.0)	10.9 - 22.8
Marital status, n (%)	Has partner	118	17 (14.4)	9.1 - 22.0
	No partner	155	29 (18.7)	13.3 - 25.7
Education status, n (%)	primary & below	160	30 (18.8)	13.4 - 25.6
	Secondary & above	113	16 (14.2)	8.8 - 22.0
Employment status, n (%)	Unemployed	126	30 (23.8)	17.1 - 32.1
	Employed	147	16 (10.9)	6.7 - 17.1
Religion, n (%)	Muslim	45	10(22.2)	12.3 - 36.8
	Catholic	88	12(13.6)	7.9 - 22.6
	Anglican	96	12(12.5)	7.2 - 20.8
	Pentecostal	35	10(28.6)	15.9 - 45.8
	Other^*^	9	2(22.2)	5.1 - 60.4
Own a house, n (%)	No	91	14(15.4)	9.3 - 24.4
	Yes	182	32(17.6)	12.7 - 23.9
Opportunistic infections^**^, n (%)	No	252	42(16.7)	12.5 - 21.8
	Yes	21	4(19.0)	7.1 - 41.9
Current Viral load, n (%)	Suppressed	267	45(16.9)	12.8 - 21.9
	Unsuppressed	6	1(16.7)	1.8 - 68.0
Current WHO Stage, n (%)	Stage1	244	40(16.4)	12.2 - 21.6
	Stage2	20	3(15.0)	4.7 - 38.5
	Stage3	9	3(33.3)	10.3 - 68.6
Current ART regimen, n (%)	First line	249	40(16.1)	12.0 - 21.2
	Second line	22	4(18.2)	6.8 - 40.4
	Third line	2	2(100)	
Duration of Current regimen, n (%)	Less than 3 years	122	25(20.5)	14.2 - 28.7
	3 years & above	151	21(13.9)	9.2 - 20.4
Has a comorbidity^¶^, n (%)	No	162	28(17.3)	12.2 - 24.0
	Yes	111	18(16.2)	10.4 - 24.4
Alcohol consumption, n (%)	No	216	38(17.6)	13.0 - 23.3
	Yes	57	8(14.0)	7.1 - 25.8
Smoking, n (%)	No	261	43(16.5)	12.4 - 21.5
	Yes	12	3(25.0)	7.8 - 56.8
HIV Disclosure status, n (%)	No	20	3(15.0)	4.7 - 38.5
	Yes	253	43(17.0)	12.8 - 22.2
Has a treatment supporter, n (%)	Yes	157	19(12.1)	7.8 - 18.2
	No	116	27(23.3)	16.4 - 31.9
Had a previous history of mental health disorder, n (%)	No	253	42(16.6)	12.5 - 21.7
	Yes	20	4(20.0)	7.5 - 43.6
Adherence, n (%)	Less than 2 doses	235	40(17.0)	12.7 - 22.4
	2 or more doses	38	6(15.8)	7.2 - 31.3
Family history of mental health disorder, n (%)	No	218	29(13.3)	9.4 - 18.5
	Yes	55	17(30.9)	20.0 - 44.4
Has Stigma, n (%)	No	234	29(12.4)	8.7–17.3
	Yes	39	17(43.6)	28.9 - 59.5
Family support, n (%)	Inadequate	72	25(34.7)	24.6 - 46.5
	Adequate	201	21(10.4)	6.9 - 15.5

# Overall, prevalence of probable anxiety disorders among the 273 participants,

* Others (Seventh day Adventists, Traditionalists), WHO- World Health Organization, ART- Anti-Retroviral Treatment

** Opportunistic infections (Tuberculosis, Cryptococcal meningitis, syphilis, and Kaposi’s sarcoma),

¶ comorbidity (HTN-Hypertension, DM-Diabetes Mellitus, CVD-Cardiovascular Disease, TB-Tuberculosis), HIV-Human Immunodeficiency Virus, CI-confidence Interval

The prevalence of probable anxiety disorders was similar among all categories of clinical characteristics Although, the prevalence of probable anxiety disorders among those with WHO stage 3 33.3%, 95 (CI: 10.3–68.6) was seemingly higher than other WHO stages, it was not significantly different. Participants on third-line ART regimen (100%) have higher prevalence of probable anxiety disorders than those on other regimen lines. Those who smoke 25% (95% CI 7.8–56.8) have higher prevalence than nonsmokers. (Table 2)

The prevalence of probable anxiety disorders was relatively similar among all categories of psychological characteristics. However, the prevalence was higher among participants with a family history of mental health disorder (30.9%, 95% CI 20.0–44.4) compared to those without. The prevalence was higher among those with stigma (43.6%, 95% CI 28.9–59.5) and prevalence was higher among those with inadequate family support (34.7%, 95% CI 24.6–46.5) compared to those with adequate family support as indicated in Table 2.

### Factors associated with probable anxiety disorders

In the bivariate analysis, employment status (cPR = 2.188, 95%CI: 1.251,3.826), Duration on the current ART regimen (cPR = 1.473, 95%CI: 0.867,2.503, Treatment supporter (cPR = 1.923, 95%CI: 1.125,3.289), Family history of mental health disorder (cPR = 2.324, 95%CI: 1.379,3.914), stigma (cPR = 3.517, 95%CI: 2.145,5.766) and family support (cPR = 3.323, 95%CI: 1.986,5.561) were significantly associated with probable anxiety disorder. Furthermore, in multivariate analysis employment status (aPR = 2.113, 95%CI: 1.252,3.567), family history of mental health disorder (aPR = 2.041 95%CI: 1.228,3.394), stigma (aPR = 2.564 95%CI: 1.544, 4.257) and family support (aPR = 2.169 95%CI: 1.272,3.699) were significantly associated with probable anxiety disorder (Table 3)

**Table 3:** Factors associated with probable anxiety disorders among elderly PLWH, N = 273.

Variable	Category	cPR	95% CI	P-value	aPR	95% CI	P-value
Age	56 & above	Ref	‐	‐	‐	‐	‐
	less than 56	1.009	0.594, 1.715	0.973	1.051	0.628,1.760	0.849
Sex	Female	Ref	‐	‐	‐	‐	‐
	Male	1.118	0.659, 1.896	0.679	1.360	0.816,2.265	0.238
Marital status	Has partner	Ref	‐	‐	‐	‐	‐
	No partner	1.299	0.749, 2.250	0.351	‐	‐	‐
Education status	≥Secondary	Ref	‐	‐	‐	‐	‐
	≤primary	1.324	0.758, 2.313	0.324	‐	‐	‐
Employment status	Employed	Ref	‐	‐	Ref	‐	‐
	Unemployed	2.188	1.251,3.826	**0.006**	2.198	1.272,3.798	**0.005**
Religion	Pentecostal	Ref	‐	‐	‐	‐	‐
	Muslim	0.778	0.364, 1.661	0.516	‐	‐	‐
	Catholic	0.477	0.227, 1.004	**0.051**	‐	‐	‐
	Anglican	0.438	0.207, 0.923	**0.03**	‐	‐	‐
	Other^*^	0.778	0.205,2.947	0.712	‐	‐	‐
Own a house	No	Ref	‐	‐	‐	‐	‐
	Yes	1.143	0.642, 2.034	0.65	‐	‐	‐
Opportunistic infections	No	Ref	‐	‐	‐	‐	‐
	yes	1.143	0.453,2.884	0.777	‐	‐	‐
Current viral load	Unsuppressed	Ref	‐	‐	‐	‐	‐
	suppressed	1.011	0.165, 6.193	0.99	‐	‐	‐
Current WHO stage	Stage3	Ref	‐	‐	‐	‐	‐
	Stage2	0.45	0.111,1.818	0.262	‐	‐	‐
	Stage1	0.492	0.187, 1.295	0.151	‐	‐	‐
Current ART regimen	First line	Ref	‐	‐	‐	‐	‐
	Second line	1.132	0.445, 2.876	0.795	‐	‐	‐
	Third line	6.225	4.684, 8.273	**<0.001**	‐	‐	‐
Duration on current regimen	≥3 years	Ref	‐	‐	‐	‐	‐
	<3 years	1.473	0.867,2.503	**0.152**	‐	‐	‐
Has a comorbidity^¶^	Yes	Ref	‐	‐	‐	‐	‐
	No	1.066	0.620,1.832	0.818	‐	‐	‐
Alcohol consumption	Yes	Ref	‐	‐	‐	‐	‐
	No	1.253	0.619,2.538	0.53	‐	‐	‐
Smoking	No	Ref	‐	‐	‐	‐	‐
	Yes	1.517	0.548,4.205	0.423	‐	‐	‐
HIV disclosure status	No	Ref	‐	‐	‐	‐	‐
	Yes	1.133	0.385,3.337	0.821	‐	‐	‐
Has treatment supporter	Yes	Ref	‐	‐	‐	‐	‐
	No	1.923	1.125,3.289	**0.017**	‐	‐	‐
Previous history of mental health disorder	No	Ref	‐	‐	‐	‐	‐
	Yes	1.205	0.480,3.025	0.692	‐	‐	‐
Adherence	≥2	Ref	‐	‐	‐	‐	‐
	<2	1.078	0.490,2.371	0.852	‐	‐	‐
Family history of mental health disorder	No	Ref	‐	‐	Ref	‐	‐
	Yes	2.324	1.379,3.914	**0.002**	1.981	1.265,3.371	**0.012**
Has Stigma	No	Ref	‐	‐	Ref	‐	‐
	Yes	3.517	2.145,5.766	**<0.001**	2.644	1.570,4.452	**<0.001**
Family support	Adequate	Ref	‐	‐	Ref	‐	‐
	Inadequate	3.323	1.986,5.561	**<0.001**	2.145	1.233,3.733	**0.007**

* Others (Seventh day Adventists, Traditionalists), WHO- World Health Organization, ART- Anti-Retroviral Treatment, **Opportunistic infections (Tuberculosis, Cryptococcal meningitis, syphilis, and Kaposi’s sarcoma),

¶ comorbidity (HTN-Hypertension, DM-Diabetes Mellitus, CVD-Cardiovascular Disease, TB-Tuberculosis), HIV- Human Immunodeficiency Virus, CI-confidence Interval, cPR-crude Prevalence ratio, aPR-Adjusted Prevalence Ratio.

## Discussion

The overall prevalence of probable anxiety disorders in this cross-sectional study was found to be 16.8% (95% CI 12.5–21.6) as shown in Table 2. This means that one in every six elderly people living with HIV experienced anxiety disorders. These findings are higher than the 9.1% reported in Buikwe district, Uganda [24]. This could be because the study was conducted in the Buikwe district a rural setting. There is a difference in the cost of living between the rural and urban areas. A community-based cross-sectional study in India found a prevalence of anxiety disorders among the elderly to be 10.7% [25], a prevalence that is lower than the 16.8% in this study. This could be because their study was done among a general population aged 60 years and above in a rural setting and did not take into account the HIV serostatus. However, the findings are lower than the 25.6% reported in Ethiopia [26]. This could be because the study by Kechine was conducted in multiple sites and the mean age was 37.5 (8.68) years. These findings were also slightly lower than the 17.1% reported by Kirmizioglu in Turkey [8]. This could have been because of the difference in tools used whereby Structured Clinical Interview for DSM-IV Axis 1 Disorder (SCID-1) was used in the study conducted in Turkey.

The prevalence reported in this study could be an underestimate because of self-reporting as there could be some fears to disclose. However, the findings are considered true for those participants who reported having anxiety disorders because the GAD-7 tool had a reliability coefficient of 0.92 in the study and participants had to respond to other questions for more details. These study findings imply that there’s a need for intensified screening for anxiety disorders among elderly PLWH.

The findings from our study show a significant association between probable anxiety disorders and employment status among elderly persons living with HIV. As shown in Table 3 the adjusted prevalence ratio (aPR) of 2.113 indicates that unemployed elderly persons living with HIV are 2.1 times more likely to have anxiety disorders compared to those who are employed. These findings are in agreement with those reported by Olagunju in Nigeria [27]. Unemployment can lead to stress, financial instability, and social isolation, which can contribute to anxiety [28,29]. These findings imply that the state of being unemployed is strongly linked to probable anxiety disorders and therefore need to provide mental health support to the unemployed.

The results indicate a statistically significant association between family history of mental health disorders and probable anxiety disorders among elderly people living with HIV. As shown in Table 3, the adjusted prevalence ratio (aPR) of 2.041 indicates that individuals with familial history of mental health disorders are twice more likely to have anxiety disorders than those without familial history. All types of mental illness tend to run in families, and the risk of developing an illness is associated with the degree of biological relatedness to the affected individual [30,31]. Growing up in a family where mental health issues are present might influence an individual’s own mental health, possibly due to shared environmental factors or learned behaviors [32]. These findings imply that every elderly person with family history of any mental health disorder needs to be screened for anxiety disorders.

The results indicate a highly significant association between stigma and probable anxiety disorders among the elderly people living with HIV. As shown in Table 3 the adjusted prevalence ratio (aPR) of 2.564 shows that individuals with stigma are 2.6 times more likely to have anxiety disorders compared to those without stigma. These findings are in agreement with those reported in Cambodia and Ethiopia where stigma was significantly associated with anxiety [26,33]. Stigma increases stress, decreases one’s capacity to cope and limits one’s capacity to do their daily activities hence causing poor quality of life leading to anxiety [34]. The significant association between stigma and anxiety disorders underscores the need for interventions aimed at promoting social support, acceptance and inclusivity to mitigate the negative effects of stigma on mental health.

The results indicate a statistically significant association between family support and probable anxiety disorders among elderly people living with HIV. As shown in Table 3, the adjusted prevalence ratio of 2.169 suggests that individuals with inadequate family support are 2.2 times more likely to have probable anxiety disorders compared to those with adequate family support. These findings are in agreement with those reported in Almeria, Spain and in Nigeria [35,36]. Family conflict and a lack of warmth and affection can prevent individuals in the family from expressing their emotions which can cause anxiety disorders [34]. These results imply that lack of attachment and emotional warmth leads to anxiety disorders.

## Limitations of the study

This study did not use a diagnostic tool administered by a mental health professional to confirm the presence of any type of anxiety disorder. Screening tools like theGAD-7 can help identify individuals who may be at risk for anxiety disorders, but they are not diagnostic tools. These tools are designed to be quick, self-reported questionnaires that flag symptoms indicative of anxiety but do not replace the more comprehensive diagnostic process conducted by a trained mental health professional. Formal diagnosis of anxiety disorders generally requires a structured clinical interview and possibly other diagnostic assessments conducted by a qualified professional, such as a psychologist or psychiatrist. It is against this background that we use the term probable anxiety disorder [37].

The study could have had some potential information bias due to self-reporting of anxiety using the GAD-7 tool, stigma using the 13-stigma scale and family support using the family support scale for the elderly. This could also have resulted in an underestimate of the prevalence of anxiety disorders.

The study also could have suffered random error due to inadequate sample size that resulted in wide confidence intervals of some variables. still, due to the small sample size, there may have been inadequate power to detect association, especially for variables with categories that had low numbers.

There could have been some confounding as not all variables were assessed and analyzed and these included being on other medication, gender-based-violence and residential setting among others.

The study was not able to assess alcohol use using a standard tool to do quantification as it focused on knowing if an individual used alcohol or not. The amount of alcohol used and the frequency may have a relationship with anxiety.

The temporal relationship could not be ascertained as well as the causal relationship.

The results of the study can be generalizable to urban-based public facilities in Uganda which may not be the case in other parts of the country.

However, the above limitations and biases highlighted did not affect the study substantially hence the findings are valid

## Conclusion and recommendations

The study found that one in every six elderly persons living with HIV had probable anxiety disorders. Unemployment, family history of mental illness, stigma, and inadequate family support were identified as factors associated with probable anxiety disorders among elderly persons with HIV. These findings suggest the need for the integrated mental health screening in HIV care, economic empowerment programs, family support initiatives, and stigma-reduction campaigns among people living with HIV. Furthermore, a longitudinal study is recommended to assess how these factors influence the mental health of elderly persons with HIV over time.

## Supporting information

S1 File STROBE checklist. (DOC)

S2 File Supplementary file (Questionnaire). (DOCX)

S3 File Dataset file. (XLS)

## References

[1] Global HIV & AIDS statistics — Fact sheet [press release]. 2021.

[2] UNAIDS. The Global HIV/AIDS Epidemic. 2021.

[3] Autenrieth CS, Beck EJ, Stelzle D, Mallouris C, Mahy M, Ghys P. Global and regional trends of people living with HIV aged 50 and over: Estimates and projections for 2000-2020. PLoS One. 2018;13(11):e0207005. doi: 10.1371/journal.pone.0207005 30496302 PMC6264840

[4] MOH. Facts on HIV and AIDS in Uganda 2021 (Based on data ending 31st December 2020). 2021.

[5] MOH. Facts on HIV and AIDS in Uganda. Uganda AIDS Commission secretariate: Ministry of health; 2023.

[6] Rueda S, Law S, Rourke SB. Psychosocial, mental health, and behavioral issues of aging with HIV. Curr Opin HIV AIDS. 2014;9(4):325–31. doi: 10.1097/COH.0000000000000071 24824890

[7] Han J, Jia P, Huang Y, Gao B, Yu B, Yang S, et al. Association between social capital and mental health among older people living with HIV: the Sichuan Older HIV-Infected Cohort Study (SOHICS). BMC Public Health. 2020;20(1):581. doi: 10.1186/s12889-020-08705-6 32345273 PMC7189431

[8] Kirmizioglu Y, Doğan O, Kuğu N, Akyüz G. Prevalence of anxiety disorders among elderly people. Int J Geriatr Psych. 2009;24(9):1026–33. doi: 10.1002/gps.2215 19259977

[9] Mpondo BCT. HIV Infection in the elderly: arising challenges. J Aging Res. 2016;2016:2404857. doi: 10.1155/2016/2404857 27595022 PMC4993911

[10] UNAIDS. People aged 50 years and older. UNAIDS. 2014. https://www.unaids.org/sites/default/files/media_asset/12_Peopleaged50yearsandolder.pdf

[11] Fatti G, Ngorima-Mabhena N, Mothibi E, Muzenda T, Choto R, Kasu T, et al. Outcomes of three- versus six-monthly dispensing of antiretroviral treatment (ART) for stable HIV patients in community ART refill groups: a cluster-randomized trial in Zimbabwe. J Acquir Immune Defic Syndr. 2020;84(2):162–72. doi: 10.1097/QAI.0000000000002333 32097252 PMC7172979

[12] Pourhoseingholi MA, Vahedi M, Rahimzadeh M. Sample size calculation in medical studies. Gastroenterol Hepatol Bed Bench. 2013;6(1):14–7. 24834239 PMC4017493

[13] Kish L. Sampling organizations and groups of unequal sizes. Am Sociol Rev. 1965;30:564–72. 14325826

[14] Opio JN, Munn Z, Aromataris E. Prevalence of mental disorders in Uganda: a systematic review and meta-analysis. Psych Quart. 2022;93(1):199–226. 10.1007/s11126-021-09941-8 34427855

[15] Spitzer RL, Kroenke K, Williams JBW, Löwe B. A Brief Measure for Assessing Generalized Anxiety Disorder: The GAD-7. Arch Internal Med. 2006;166(10):1092–7. 16717171 10.1001/archinte.166.10.1092

[16] Kroenke K, Spitzer RL, Williams JB, Monahan PO, Löwe B. Anxiety disorders in primary care: prevalence, impairment, comorbidity, and detection. Ann Intern Med. 2007;146(5):317–25. 17339617 10.7326/0003-4819-146-5-200703060-00004

[17] Akena D, Kiguba R, Muwhezi WW, Kwesiga B, Kigozi G, Lukwata H, et al. The prevalence and factors associated with mental disorders in a community setting in central Uganda. PLoS One. 2023;18(5):e0285091. doi: 10.1371/journal.pone.0285091 37141327 PMC10159349

[18] Mwangala PN, Nasambu C, Wagner RG, Newton CR, Abubakar A. Prevalence and factors associated with mild depressive and anxiety symptoms in older adults living with HIV from the Kenyan coast. J Int AIDS Soc. 2022;25 Suppl 4(Suppl 4):e25977. doi: 10.1002/jia2.25977 36176027 PMC9522642

[19] Emlet CA. Measuring stigma in older and younger adults with HIV/AIDS: an analysis of an HIV stigma scale and initial exploration of subscales. Research on Social Work Practice. 2005;15(4):291–300.

[20] Uddin M, Bhuiyan A. Development of the family support scale (FSS) for elderly people. MOJ Gerontol Geriatrics. 2019;4(1):17–20.

[21] McNutt LA, Wu C, Xue X, Hafner JP. Estimating the relative risk in cohort studies and clinical trials of common outcomes. 2003. 10.1093/aje/kwg074 12746247

[22] Coutinho LM, Scazufca M, Menezes PR. Methods for estimating prevalence ratios in cross-sectional studies. Rev Saude Publica. 2008. 19009156

[23] Barros AJD, Hirakata VN. Alternatives for logistic regression in cross-sectional studies: an empirical comparison of models that directly estimate the prevalence ratio. BMC Med Res Methodol. 2003;3:21. doi: 10.1186/1471-2288-3-21 14567763 PMC521200

[24] Mugisha J, Byansi PK, Kinyanda E, Bbosa RS, Damme TV, Vancampfort D. Moderate to severe generalized anxiety disorder symptoms are associated with physical inactivity in people with HIV/AIDS: a study from Uganda. Int J STD AIDS. 2021;32(2):170–5. doi: 10.1177/0956462420942992 33323069

[25] Nair SS, Raghunath P, Nair SS. Prevalence of psychiatric disorders among the rural geriatric population: a pilot study in Karnataka, India. Cent Asian J Glob Health. 2015;4(1):138. doi: 10.5195/cajgh.2015.138 29138712 PMC5661194

[26] Kechine T, Ali T, Worku T, Abdisa L, Assebe Yadeta T. Anxiety and associated factors among clients on highly active antiretroviral therapy (HAART) in Public hospitals of southern ethiopia: a multi-center cross-sectional study. Psychol Res Behav Manag. 2022;15:3889–900. doi: 10.2147/PRBM.S385630 36605175 PMC9809403

[27] Olagunju AT, Adeyemi JD, Ogbolu RE, Campbell EA. A study on epidemiological profile of anxiety disorders among people living with HIV/AIDS in a sub-Saharan Africa HIV clinic. AIDS Behav. 2012;16(8):2192–7. doi: 10.1007/s10461-012-0250-x 22772942

[28] Arena AF, Mobbs S, Sanatkar S, Williams D, Collins D, Harris M, et al. Mental health and unemployment: a systematic review and meta-analysis of interventions to improve depression and anxiety outcomes. J Affect Disord. 2023;335:450–72. doi: 10.1016/j.jad.2023.05.027 37201898

[29] Ryu S, Fan L. The relationship between financial worries and psychological distress among U.S. adults. J Fam Econ Issues. 2023;44(1):16–33. doi: 10.1007/s10834-022-09820-9 35125855 PMC8806009

[30] Rasic D, Hajek T, Alda M, Uher R. Risk of mental illness in offspring of parents with schizophrenia, bipolar disorder, and major depressive disorder: a meta-analysis of family high-risk studies. Schizophr Bull. 2014;40(1):28–38. doi: 10.1093/schbul/sbt114 23960245 PMC3885302

[31] Gottesman II, Laursen TM, Bertelsen A, Mortensen PB. Severe mental disorders in offspring with 2 psychiatrically ill parents. Arch Gen Psychiatry. 2010;67(3):252–7. doi: 10.1001/archgenpsychiatry.2010.1 20194825

[32] WHO. Social determinants of mental health. Foundation WHOatC, ed. Geneva: World Health Organisation; 2014.

[33] Yi S, Chhoun P, Suong S, Thin K, Brody C, Tuot S. AIDS-related stigma and mental disorders among people living with HIV: a cross-sectional study in Cambodia. PLoS One. 2015;10(3):e0121461. doi: 10.1371/journal.pone.0121461 25806534 PMC4373790

[34] Zhang Y, Chai C, Xiong J, Zhang L, Zheng J, Ning Z. The impact of anxiety, depression, and social support on the relationship between HIV-related stigma and mental health-related quality of life among Chinese patients: a cross-sectional, moderate-mediation study. BMC Psych. 2023;23(1):818. 10.1186/s12888-023-05103-1 PMC10634100 37940853

[35] Barragán Martín AB, Molero Jurado MdM, Pérez-Fuentes MdC, Oropesa Ruiz NF, Martos Martínez Á, Simón Márquez MdM. Int J Environ Res Public Health. 2021;18(10):5145. doi: 10.3390/ijerph18105145 34066285 PMC8152060

[36] Olagunju AT, Adeyemi JD, Erinfolami AR, Ogundipe OA. Factors associated with anxiety disorders among HIV-positive attendees of an HIV clinic in Lagos, Nigeria. Int J STD AIDS. 2012;23(6):389–93. doi: 10.1258/ijsa.2011.011200 22807530

[37] Leichsenring F, Abbass A, Fonagy P, Levy KN, Lilliengren P, Luyten P, et al. WHO treatment guideline for mental disorders. Lancet Psych. 2024;11(9):676–7. doi: 10.1016/S2215-0366(24)00169-X 39067469

