# Supplementary material for: Prevalence and factors associated with probable anxiety disorders among elderly persons living with HIV at Mulago ISS clinic: A cross-sectional study
Source: PLoS One. 2025 Aug 1;20(8):e0329111. doi: 10.1371/journal.pone.0329111 (PMC12316274; doi:10.1371/journal.pone.0329111)
Supplement: S2 File — (DOCX) [file pone.0329111.s002.docx]

***APPENDIX 2: Study Questionnaire in English.***

**Study title:** Prevalence and factors associated with probable anxiety disorders among elderly PLHIV at ISS clinic Mulago.

| Unique participant code | | |  | |
| --- | --- | --- | --- | --- |
| **Data collector information** | | | | |
| Study staff initial | | |  | |
| Date of collection | | |  | |
| **Section A: Generalised Anxiety disorder 7-item (GAD-7)** | | | | |
| Over the **last 2 weeks**, how often have you been bothered by the following problems? | | | | |
| QUESTIONS | Not at all | Several days | More than half the days | Nearly everyday |
| 1. Fealing nervous, anxious or on edge | 0 | 1 | 2 | 3 |
| 1. Not being able to stop or control worrying | 0 | 1 | 2 | 3 |
| 1. Worrying too much about different things | 0 | 1 | 2 | 3 |
| 1. Trouble relaxing | 0 | 1 | 2 | 3 |
| 1. Being so restless that it is hard to sit still | 0 | 1 | 2 | 3 |
| 1. Becoming easily annoyed or irritable | 0 | 1 | 2 | 3 |
| 1. Feeling afraid as if something awful might happen | 0 | 1 | 2 | 3 |
| Total |  | | | |
| If you checked off any problems, how difficult have these problems made it for you to: do your work, take care of things at home, or get along with other people | 1= Not difficult at all | 2= Somewhat difficult | 3= Very difficult | 4= Extremely difficult |

| 1. | Unique participant code |  |
| --- | --- | --- |
|  | **Data collector information** |  |
| 2. | Study staff initial |  |
| 3. | Date of collection |  |
|  | **Section B: Demographic characteristics** |  |
| 4. | Age in completed years | **………………………………………..** |
| 5. | Sex | - Male - Female |
| 6. | Employment status | - Employed - Unemployed |
| 7. | What is your source of income? | …………………………………………………… |
| 8. | What is your monthly income? | …………………………………………………. |
| 9. | What is your Marital status? | - Married - Cohabiting - Separated - Single - Widow/widower |
| 10. | What is the highest Level of education? | - No formal education - Primary - Secondary - Tertiary |
| 11. | What is your religion? | - Muslim - Catholic - Anglican - Other |
| 12. | Do you live in your own house? | - Yes - No |
|  | **Section C: Clinical characteristics** |  |
| 13. | Is there any underlying opportunistic infection? | - Yes - No |
| 14. | What is the current Viral load? |  |
| 15. | What is the current WHO staging? | - Stage I - Stage II - Stage III - Stage IV |
| 16. | Current ART Regimen | - First line - Second line - Third line |
| 17. | Duration on current ART regimen? |  |
| 18. | Underlying comorbidities | - Hypertension - Diabetes Mellitus - Tuberculosis - Cardiovascular diseases - Other……………(specify) |
| 19. | Do you drink alcohol | - Yes - No |
| 20. | Do you smoke? | - Yes - No |
|  | **Section D: Psychological characteristics** |  |
| 21. | Have you disclosed your HIV status to anyone? | - Yes - No |
| 22. | Who is your treatment Supporter? | - Spouse - Relatives - A friend - None |
| 23. | Any previous history of mental health disorder? | - Yes - No |
| 24. | What is the number of missed doses in a month? |  |
| 25. | Family history of mental health disorder? | - Yes - No |

SHORT HIV STIGMA SCALE

|  | item | Strongly agree | Agree | Neither Agree nor disagree | Disagree | Strongly disagree |
| --- | --- | --- | --- | --- | --- | --- |
| 1. | I felt blamed by others for my illness. | 1 | 2 | 3 | 4 | 5 |
| 2. | I felt ashamed of my illness. | 1 | 2 | 3 | 4 | 5 |
| 3. | I thought my illness was a punishment for things I’ve done in the past. | 1 | 2 | 3 | 4 | 5 |
| 4. | I feared that I might lose my job if someone found out about my illness. | 1 | 2 | 3 | 4 | 5 |
| 5. | I felt compelled to change my residence because of my illness. | 1 | 2 | 3 | 4 | 5 |
| 6. | I avoided getting treatment because someone might find out about my illness. | 1 | 2 | 3 | 4 | 5 |
| 7. | I feared that people would hurt my family if they learned about my illness. | 1 | 2 | 3 | 4 | 5 |
| 8. | I thought other people were uncomfortable being with me. | 1 | 2 | 3 | 4 | 5 |
| 9. | I felt people avoid me because of my illness. | 1 | 2 | 3 | 4 | 5 |
| 10. | I feared I would lose my friends if they learned about my illness. | 1 | 2 | 3 | 4 | 5 |
| 11. | I feared my family would reject me if they learned about my illness. | 1 | 2 | 3 | 4 | 5 |
| 12. | I felt I wouldn’t get as good health care if people knew about my illness. | 1 | 2 | 3 | 4 | 5 |
| 13. | People who know I am HIV positive treat me with kid gloves | 1 | 2 | 3 | 4 | 5 |

**Family Support Scale (FSS) for elderly people**

|  | ITEM | NO | LITTLE | SOME | MUCH |
| --- | --- | --- | --- | --- | --- |
| 1 | My family loves me | 0 | 1 | 2 | 3 |
| 2 | I get respect from my family | 0 | 1 | 2 | 3 |
| 3 | My family helps me with daily activities | 0 | 1 | 2 | 3 |
| 4 | My family helps me with religious activities | 0 | 1 | 2 | 3 |
| 5 | My family gives me useful information | 0 | 1 | 2 | 3 |
| 6 | My family give me emotional support | 0 | 1 | 2 | 3 |
| 7 | My family shares important decisions with me | 0 | 1 | 2 | 3 |
| 8 | My family understands my personal desires | 0 | 1 | 2 | 3 |
| 9 | My family helps me to participate in social events | 0 | 1 | 2 | 3 |
| 10 | My family listens my problems | 0 | 1 | 2 | 3 |
| 11 | My family helps to solve my problems | 0 | 1 | 2 | 3 |
| 12 | My family is aware of my health | 0 | 1 | 2 | 3 |
| 13 | My family helps in my treatment | 0 | 1 | 2 | 3 |
| 14 | My family treats me as an important person | 0 | 1 | 2 | 3 |
| 15 | My family gives me money when I need it | 0 | 1 | 2 | 3 |
| 16 | My family is careful about my food | 0 | 1 | 2 | 3 |
| 17 | My family is careful about my sleep | 0 | 1 | 2 | 3 |
| 18 | My family gives me companionship | 0 | 1 | 2 | 3 |
| 19 | My family helps me to stay happy | 0 | 1 | 2 | 3 |
| 20 | I am satisfied with my family's support | 0 | 1 | 2 | 3 |
